# Performance of Ag-ELISA in the diagnosis of *Taenia solium* cysticercosis in naturally infected pigs in Tanzania

**DOI:** 10.1186/s13071-020-04416-4

**Published:** 2020-10-27

**Authors:** Mwemezi L. Kabululu, Maria V. Johansen, James E. D. Mlangwa, Ernatus M. Mkupasi, Uffe C. Braae, Chiara Trevisan, Angela Colston, Claudia Cordel, Marshall W. Lightowlers, Helena A. Ngowi

**Affiliations:** 1 Tanzania Livestock Research Institute (TALIRI)-Uyole, Mbeya, Tanzania; 2 Department of Veterinary and Animal Sciences, Faculty of Health and Medical Sciences, University of Copenhagen, Frederiksberg, Denmark; 3 Department of Veterinary Medicine and Public Health, College of Veterinary Medicine and Biomedical Sciences, Sokoine University of Agriculture, Morogoro, Tanzania; 4 Department of Infectious Disease Epidemiology, Statens Serum Institut, Copenhagen, Denmark; 5 One Health Center for Zoonoses and Tropical Veterinary Medicine, Ross University School of Veterinary Medicine, Basseterre, Saint Kitts and Nevis; 6 Department of Biomedical Sciences, Institute of Tropical Medicine, Antwerp, Belgium; 7 Global Alliance for Livestock Veterinary Medicines (GALVmed), Nairobi, Kenya; 8 Global Alliance for Livestock Veterinary Medicines (GALVmed), Bloemfontein, Free State South Africa; 9 UVet, Faculty of Veterinary and Agricultural Sciences, University of Melbourne, Werribee, VIC Australia

**Keywords:** Ag-ELISA, Diagnosis, *Taenia solium*, Pigs, Tanzania

## Abstract

**Background:**

*Taenia solium* is a zoonotic parasite responsible for neurocysticercosis—a major cause of late-onset acquired epilepsy in humans. Lack of affordable, specific and sensitive diagnostic tools hampers control of the parasite. This study assessed the performance of an antigen detection enzyme-linked immunosorbent assay (Ag-ELISA) in the diagnosis of viable *T. solium* cysticercosis in naturally infected slaughter-age pigs in an endemic area in Tanzania.

**Methods:**

A total of 350 pigs were bled before they were slaughtered and their carcases examined. Serum was analyzed for circulating antigens by using a monoclonal antibody-based B158/B60 Ag-ELISA. Each carcase was examined for the presence of *Taenia hydatigena* cysticerci and half carcase musculature together with the whole brain, head muscles, tongue, heart and diaphragm were sliced with fine cuts (< 0.5 cm) to reveal and enumerate *T. solium* cysticerci. Half carcase dissection can detect at least 84% of infected pigs. Prevalence and their 95% confidence intervals (CI) were calculated in Stata 12. Sensitivity, specificity, predictive values and likelihood ratios were determined.

**Results:**

Twenty–nine pigs (8.3%, 95% CI: 5.6–11.7%) had viable *T. solium* cysticerci while 11 pigs had *T. hydatigena* cysticerci (3.1%, 95% CI: 1.6–5.5%). No co-infection was observed. Sixty-eight pigs (19.4%, 95% CI: 15.4–20%) tested positive on Ag-ELISA; of these, 24 had *T. solium* cysticerci and 7 had *T. hydatigena* cysticerci. Sensitivity and specificity were determined to be 82.7% and 86.3%, respectively. Positive and negative predictive values were 35.2% and 98.2%, respectively. Likelihood ratios for positive and negative Ag-ELISA test results were 6.0 and 0.2, respectively. There was a significant positive correlation between the titre of circulating antigens and intensity of *T. solium* cysticerci (*r*_(348)_ = 0.63, *P* < 0.001).

**Conclusions:**

The Ag-ELISA test characteristics reported in this study indicate that the test is more reliable in ruling out *T. solium* cysticercosis in pigs, than in confirming it. Hence, a negative result will almost certainly indicate that a pig has no infection, but a positive result should always be interpreted with caution. Estimates of *T. solium* prevalence based on Ag-ELISA results should, therefore, be adjusted for test performance characteristics and occurrence of *T. hydatigena*.
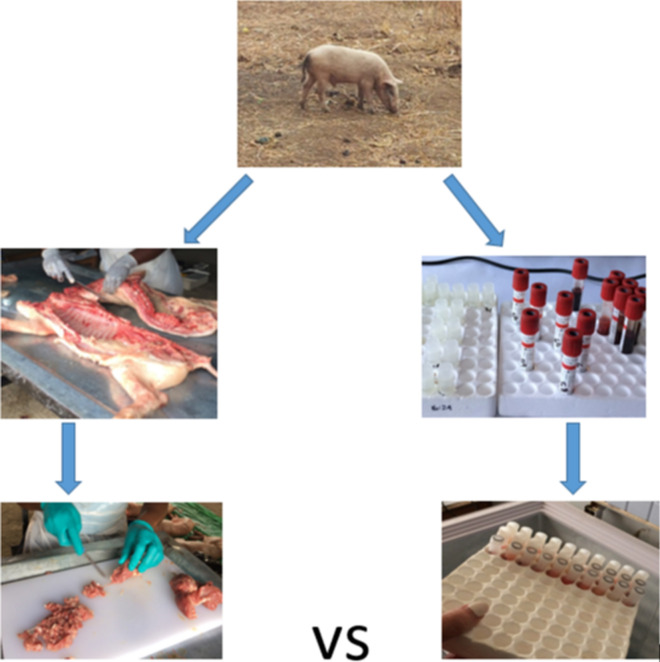

## Background

The pork tapeworm, *Taenia solium* is a neglected zoonotic parasite which is endemic in many low-income countries, including Tanzania [1]. The parasite is responsible for neurocysticercosis (NCC) - cysticercosis of the human central nervous system - which is the major cause of late-onset acquired epilepsy in endemic areas [2]. Although several tools, including diagnostic tools, are available for its control, the parasite has remained endemic in many parts of the world. Diagnosis of *T. solium* to identify transmission hotspots, estimate disease burdens and monitor the outcome of interventions is a critical aspect for the success of its control [3, 4]. However, so far, the lack of affordable, specific and sensitive diagnostic tools have hampered control efforts [4, 5].

Tongue inspection and antigen/antibody detection enzyme-linked immunosorbent assays (ELISA) are the commonly used diagnostic methods for *T. solium* in pigs. Tongue inspection is probably the most common method for field diagnosis of *T. solium* cysticercosis in endemic areas. The method is cheap and is easy to use in the field and if properly done it has specificity close to 100% [6, 7]. However, the sensitivity of tongue inspection can be as a low as 16% [8] but it varies depending on the infection intensity [6, 7, 9, 10]. Therefore, the test is useful only in areas with high endemicity.

B158/B60 and HP10 monoclonal antibody-based Ag-ELISAs are the most common serological diagnostic tools for the diagnosis of porcine cysticercosis in research studies [6, 11]. Diagnosis can be achieved in live animals and the tests can process many samples at the same time hence suitable for use at a large scale [7, 12]. Antigen detection methods are useful in demonstrating a viable infection, unlike antibody detection methods which cannot distinguish an active infection from a mere exposure to infection, an aborted infection or a past infection [13–15]. Despite their usefulness, Ag-ELISAs are currently not readily available commercially and they require a laboratory setting including equipment and expertise, hence limiting their use to research purposes.

Using Bayesian analysis, the overall sensitivity and specificity of B158/B60 Ag-ELISA were estimated at 87% (CI: 62–98%) and 95% (CI: 90–99%), respectively [6]. Ag-ELISA has been reported to be more sensitive than tongue palpation and it is useful in the detection of light or recent infections [12, 16, 17]. However, sensitivity drops in case of lower infection intensity of viable *T. solium* cysticerci. Moreover, in areas where other *Taenia* species (such as *Taenia hydatigena*) also co-exist, specificity can drop as the assay cross-reacts with other *Taenia* species other than *T. solium* [6, 9, 18].

In view of the need for reliable diagnostic tools for the control of the *T. solium* in pigs in endemic areas, we conducted this study to evaluate the performance of B158/B60 Ag-ELISA in detecting viable *T. solium* infections in naturally infected slaughter-age pigs in an endemic area in Tanzania. Due to logistical limitations, half carcase dissection incorporating predilection sites plus half the carcase musculature was used as a reference standard. Compared to full carcase dissection, half carcase dissection is less labour-intensive and can be expected to have a sensitivity of at least 84% [19].

## Methods

### Study location

Slaughtered pigs were sourced from 16 villages, eight from each of the two districts of Mbeya Rural and Mbozi, in southwestern Tanzania, an area endemic for *T. solium*. The villages were selected based on previous studies and reports on the occurrence of *T. solium* infections. Pigs were slaughtered at the nearest public slaughter slab and carcases were transported to the Tanzania Livestock Research Institute (TALIRI), Uyole Centre, Mbeya, Tanzania, for further examinations.

### Study animals

A total of 350 pigs were included in this study, comprising of 282 slaughtered during November-December 2016 and 68 slaughtered in January 2018. The pigs were at least six months of age, apparently healthy, and representative of the pigs which would normally be slaughtered (or sold for slaughter) in the area. The pigs were purchased from randomly chosen farmers/households who consented to participate. One pig was purchased from each farmer.

### Antigen-ELISA

Pigs were bled before slaughter. Blood was obtained using a vacutainer system, by puncturing into jugular vein or cranial vena cava to let blood into plain tubes (BD vacutainer©, South Africa). Serum was separated by centrifugation at 2000 × *g* for 10 min and was dispensed into 2 ml aliquots and stored at – 20 °C before analysis. Analysis of titres of circulating antigens of *T. solium* cysticerci by Ag-ELISA was done at the regional reference laboratory at the School of Veterinary Medicine of the University of Zambia, Lusaka, Zambia.

The B158/B60 monoclonal based sandwich enzyme-linked immunosorbent assay (Ag-ELISA) was used to detect circulating antigens as described by Dorny et al. [6]. The optical densities of the samples were compared to eight known negative control sera (from Zambian pigs) at a probability (*P*) < 0.001) [20].

### Pig necropsies

Pig slaughtering followed the slaughter slab procedures. After a carcase was opened, the visceral surfaces and the entire peritoneal cavity were examined for presence of *T. hydatigena* cysticerci, paying attention to the omenta and liver surfaces [21]. Cysticerci were macroscopically identified as being *T. hydatigena* if they were relatively large (≥ 2 cm), loose hanging, translucent with a visible long-necked scolex.

Thereafter, musculature from half of a carcase was excised from bones into two portions, muscles from the forelimb and muscles from the rest of the half carcase. These muscle portions together with the whole brain, heart, tongue, head muscles and diaphragm were destined as distinct carcase sites. The carcase sites were meticulously sliced using thin cuts (< 0.5 cm) to reveal and enumerate all visible cysticerci. Cysticerci were classified as either viable (translucent fluid-filled vesicles with visible whitish scolices) or non-viable (caseous or calcified). The intensity of infection was classified as light (1–100 cysticerci), moderate (101–1000) or heavy (> 1000). A pig with at least one viable *T. solium* cysticercus in the examined carcase sites was considered positive. In case a carcase was heavily infected, a representative sample of the half carcase musculature weighing 1 kg was sliced and the number for the whole half carcase was estimated based on its weight. The total number of *T. solium* cysticerci for a pig was estimated by multiplying the unilateral (half carcase) number of cysticerci by two, plus the numbers for the brain, heart, tongue, head muscles and diaphragm.

### Data analysis

Data was entered and curated in Excel spreadsheets. The analysis was carried out using STATA© (StataCorp, 2001, Stata Statistical Software, Release 12.0. Stata Corporation 2011, College Station, TX). Frequencies and proportions were determined with their 95% confidence intervals (CI) using a binomial distribution. Sensitivity, specificity, positive and negative predictive values were calculated as conditional probabilities, according to the formulae by Thrusfield [22].

By using the Fagan’s nomogram [23], post‐test probabilities of the disease were estimated from likelihood ratios and pre-test probability (prevalence) of disease in each case of a positive and negative Ag-ELISA test result.

To assess whether there was a correlation between parasite intensity (number of viable cysticerci) and titers of circulating antigens (measured in optical densities), a non-parametric Spearman rank-order correlation was performed.

## Results

Out of 350 slaughtered pigs, viable *T. solium* cysticerci were detected in 29 pigs (8.3%, 95% CI: 5.6–11.7%). The total number of viable cysticerci ranged from 2 to 41,609 with a median of 116 cysticerci. Viable cysticerci represented about 94% of all cysticerci. Nearly all (99.8%) non-viable cysticerci were from a single pig which was heavily infected. Among the infected pigs, 13 pigs (44.8%) had light infection intensities (1–100 cysticerci); six pigs (20.7%) had moderate infection intensities (101–1000 cysticerci), and 10 (34.5%) had heavy infection intensities (> 1000 cysticerci). Eleven pigs were infected with one to two *T. hydatigena* cysticerci (3.1%, 95% CI: 1.6–5.5%). No pig was co-infected with both *T. solium* and *T. hydatigena* cysticerci.

Sixty-eight pigs (19.4%, 95% CI: 15.4–20%) tested positive on Ag-ELISA, of which 24 had *T. solium* cysticerci and 7 had *T. hydatigena* cysticerci; whereas 37 had neither of the two *Taenia* species (Table 1). Five of the 29 pigs which had *T. solium* cysticerci tested negative on Ag-ELISA and they all had light *T. solium* infection intensities (< 100). Four of the 11 pigs with *T. hydatigena* cysticerci tested negative on Ag-ELISA.

**Table 1:** Results of carcase examination for *Taenia solium* and *Taenia hydatigena* cysticerci and of B158/B60 monoclonal antibody-based antigen detecting enzyme-linked immunosorbent assay (Ag-ELISA) from 350 slaughter-age pigs in Mbeya Rural and Mbozi districts in Tanzania

Carcase examination results		Ag-ELISA result	*n*
*T. solium-*infected	*T. hydatigena-*infected		
+	+	+	0
+	−	+	24
−	+	+	7
−	−	+	37
+	−	−	5
−	+	−	4
−	−	−	273

*Abbreviations*: n, number of pigs; +, positive result; −, negative result

**Fig. 1:**
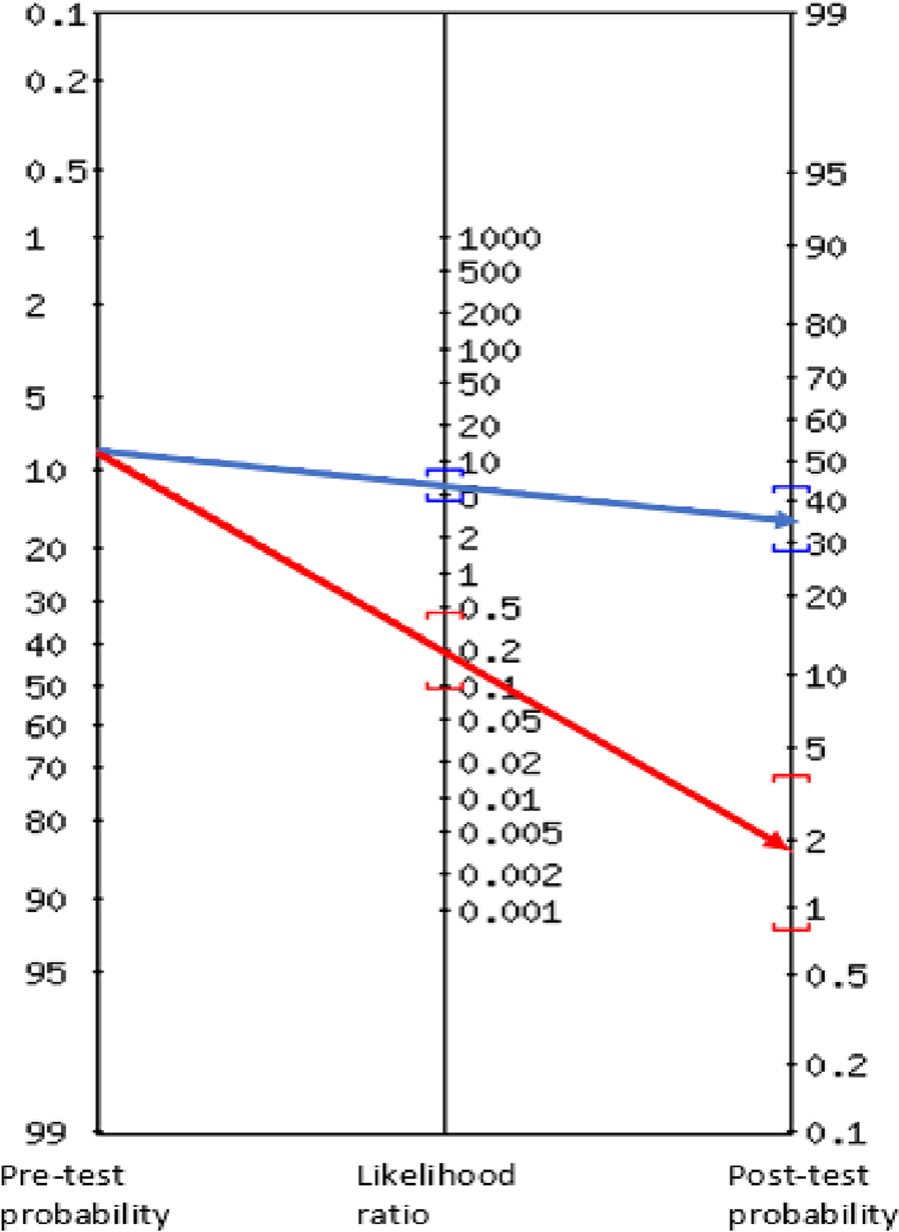
Fagan’s nomogram showing estimations of post-test probabilities of *Taenia solium* cysticercosis from likelihood ratios and pre-test probability determined from monoclonal antibody-based B158/B60 enzyme-linked immunosorbent assay (Ag-ELISA) and necropsy of 350 slaughter-age pigs of Mbeya rural and Mbozi districts in Tanzania

From the numbers presented in Table 2, B158/B60 Ag-ELISA was found to have a sensitivity of 82.7% (95% CI: 64.2–94.1%) and specificity of 86.3% (95% CI: 82–89.9%), which corresponded to false negative and false positive rates of 17.2% and 13.7%, respectively. Positive and negative predictive values were 35.3% and 98.2%, respectively. Likelihood ratios for positive and negative Ag-ELISA results were found to be 6.04 (95% CI: 4.4–8.3) and 0.2 (95% CI: 0.1–0.4), respectively. Using the Fagan’s nomogram (Fig. 1), the likelihood ratios corresponded to post-test probability of infection of 35% and 2%, for positive and negative Ag-ELISA results, respectively.

**Table 2:** Summary of the numbers of pigs infected/not infected with *Taenia solium* cysticerci that tested negative or positive on B158/B60 monoclonal antibody-based antigen detecting enzyme-linked immunosorbent assay (Ag-ELISA). These were slaughter-age pigs from Mbeya Rural and Mbozi districts in Tanzania

B158/B60 Ag-ELISA	Carcase dissection		
	Positive	Negative	Total
Positive	24	44	68
Negative	5	277	282
Total	29	321	350

There was a statistically significant correlation between the titres of circulating cysticerci antigens and the parasite intensities (*r*_(348)_ = 0.63, *P* < 0.001). However, no significant correlation was found between antigen titres of infected and non-infected pigs (*r* = 0.04, *P* = 0.83).

## Discussion

In the best possible scenario, a serological test is supposed to be highly sensitive and specific, and be able to correlate the characteristics of the infection with parasite load (see [15] for a review). Overall, the present study reports optimal sensitivity of B158/B60 Ag-ELISA in cases of infections with > 50 cysticerci but suboptimal specificity, when compared to carcase dissections, in naturally infected pigs in Tanzania.

The sensitivity and specificity estimates reported in this study were lower than what was estimated by a Bayesian method using Zambian pigs where the values were 86.7% and 94.7%, respectively [6]. A later study, also in Zambia reported a B158/B60 Ag-ELISA sensitivity of 91% to detect viable *T. solium* cysticerci, which was also higher than we report in this study [19]. However, contrary to this study, in the latter study in Zambia, full carcase dissection was performed in case no cysticerci were detected in the first carcase half. In a much recent study in Peru by Bustos et al. [24] B158/B60 Ag-ELISA showed a sensitivity 82.9% and a specificity of 96.8%, when not considering cross-reactions with *T. hydatigena*.

When assessed against the World Health Organization (WHO) Target Product Profiles (TPP) for diagnostic tests [25], the sensitivity of Ag-ELISA was 54.5% and 100% for infections with < 50 and > 50 cysticerci, respectively. These estimates were above the recommended minimum values which are 50% and 80%, respectively. However, sensitivity performed below an optimal level (70%) in case of infections with < 50 *T. solium* cysticerci. The specificity of the test was below the recommended TTP minimal value of 95%. Hence, these results have shown that the sensitivity of B158/B60 Ag-ELISA was above optimal levels in cases of infections with > 50 cysticerci but suboptimal in cases of infections with < 50 cysticerci. The results are consistent with previous studies which reported that Ag-ELISA tends to be less sensitive with lower intensity of infection [9, 19, 26].

The optimal sensitivity of B158/B60 Ag-ELISA suggests that the test could be useful in surveillance studies which intend to identify transmission hotspots of the disease in pigs for further investigations and interventions. However, because of the suboptimal specificity, the usefulness of the test in monitoring outcome of an intervention is greatly affected because of the higher rate of false positives which could indicate failure of an otherwise effective intervention. Since the test was found to be optimally sensitive, a pig with a negative test is highly unlikely to have a viable *T. solium* infection. Therefore, a negative Ag-ELISA result is more useful as it will almost certainly rule out infection. On the other hand, since the specificity of the test was found to be suboptimal, positive Ag-ELISA results do not necessarily indicate the presence of a viable infection.

The positive predictive value (PPV) estimated in this study indicated that at the reported level of prevalence of *T. solium* in the area (8.3%), the probability that an Ag-ELISA positive pig will have a viable infection is only 35.2%, suggesting that the test’s ability to confirm the infection is poor. The high negative predictive value (NPV) (98.2%) meant that the probability of an Ag-ELISA negative pig to have a viable infection is very minimal (1.8%: 1 − NPV). Therefore, a negative B158/B60 Ag-ELISA result almost certainly rules out the disease.

The reported likelihood ratio for a positive Ag-ELISA test (6.0) meant that the likelihood of a pig having *T. solium* infection increased 6-fold given a positive Ag-ELISA test result, corresponding to an increase in the probability of infection from 8.3% to 35% (Fig. 1). By using estimations suggested by McGee [27], a positive Ag-ELISA test result was, therefore, moderately suggestive of the presence of infection.

The likelihood ratio for a negative Ag-ELISA test result (0.2) meant that an Ag-ELISA negative pig was five times more likely to have no viable infection, corresponding to a decrease in the probability of infection from 8.3% to 2%. This shift in infection probability indicated that a negative Ag-ELISA test result is weakly to moderately suggestive of absence of infection.

Co-infection with *T. solium* and *T. hydatigena* was not observed in this study, consistent with the results of a previous study in the area [21]. In pigs, *T. solium* and *T. hydatigena* cysticerci are said to compete through density-dependent immune-mediated interactions such that infection with one *Taenia* species could prevent or limit infection with the other species [28]. This can be assumed to be the reason for the observed absence of co-infection. However, co-infections with *T. solium* and *T. hydatigena* have been reported in other studies in Africa [6, 19, 29] and Asia [30, 31]. Reasons for the discrepancy between results of the studies in Tanzania and elsewhere in Africa and Asia regarding co-infections with *T. solium* and *T. hydatigena* warrant further investigation.

As it has been demonstrated in previous studies [11, 18, 32–34] intensities of infection were correlated with the titres of circulating antigens in infected pigs. This implies that the titres of circulating antigens could be used as a proxy for estimating infection intensities. Hence, despite the shortcomings of Ag-ELISA in terms of sensitivity and specificity, this correlation can be useful in epidemiological and intervention studies where there is a need to estimate infection intensity in individual animals.

Cysticercal circulating antigens were detected in 37 pigs which had neither *T. solium* nor *T*. *hydatigena* cysticerci. One reason could be the possibility of a failure of infection to fully establish, as studies have shown that a significant number of cysticerci are destroyed before they mature [18, 35]. Previous studies have also shown that *T. solium* antigens can be produced well before the cysticerci are fully developed [36]. Although necropsy is considered a definitive diagnostic method for *T. solium* in pigs, it is possible that small immature cysticerci may escape detection at necropsy [37], and this could be responsible for some of the false-positive Ag-ELISA results. A study in Zambia showed that dissecting only half carcase can lead to a non-detection rate of 16% of all infected pigs [19]. As only half carcases were dissected in this study, we can assume this was partly responsible for some of the ‘false positive’ Ag-ELISA results.

Of the total of 68 pigs which were found to have cysticercal circulating antigens, seven (10.3%) had *T. hydatigena* cysticerci only. Therefore, we can assume that *T. hydatigena* can contribute at least 10% to the positive B158/B60 Ag-ELISA results in the study area. This rate could be expected to increase with an increase in the prevalence of *T. hydatigena* and should be taken into consideration when interpreting Ag-ELISA results.

As pointed out above, one major limitation of this study is that we sliced only musculature of half of the carcases. Slicing of whole carcases could have increased positive cases by approximately 16% [19]. This could have altered the test characteristics presented in this study.

## Conclusions

The test characteristics of B158/B60 Ag-ELISA reported in this study indicate that the test is more reliable in ruling out *T. solium* cysticercosis in pigs than it is in confirming it. Hence, a negative result will almost certainly indicate that a pig has no infection, but positive results should always be interpreted with caution. Estimates of *T. solium* prevalence based on Ag-ELISA results should, therefore, be adjusted for its test performance characteristics and the prevalence of *T. hydatigena*.

## Data Availability

The datasets generated and analyzed during the present study are available from the corresponding author upon a reasonable request.

## Abbreviations

Ag-ELISA: Antigen detecting Enzyme-Linked immunosorbent assay
NCC: Neurocysticercosis
CI: Confidence Interval
TALIRI: Tanzania Livestock Research Institute
CVMBS: College of Veterinary Medicine and Biomedical Sciences
SUA: Sokoine University of Agriculture
WHO: World Health Organization
TPP: Target Product Profile
